# Top Management Attributes, Psychological Capital, and Green Accounting Effectiveness in Public-Private Partnership Context

**DOI:** 10.3389/fpsyg.2019.01312

**Published:** 2019-07-30

**Authors:** Chien-Chi Chu, Yun Ji, Hsiu-Yu Lee, Yu-Ting Lin

**Affiliations:** ^1^ School of Business and Law, Foshan University, Foshan, China; ^2^ Business School of Shantou University, Shantou University, Shantou, China; ^3^ Academy of Financial Research, Business School of Wenzhou University, Wenzhou University, Zhejiang, China; ^4^ Department of Administration, Cheng Shiu University, Kaohsiung, Taiwan; ^5^ Department of Food and Beverage Management, Cheng Shiu University, Kaohsiung, Taiwan

**Keywords:** green accounting, top management, demographic attributes, psychological capital, public-private partnership

## Abstract

Through individual and/or collective psychological states affected by demographic attributes, top managers shape the corporate culture and determine the overall strategic directions of an organization. Thus, top management attributes affect the adoption of critical company-wide practices. This opinion paper discusses the implications of, and urges academic attention to, the role psychological capital plays in the relationship between top manager attributes and the effectiveness of green accounting practices adoption in a public-private partnership (PPP) context. Theoretical and practical implications are elaborated upon.

## Introduction

Researchers have applied analytic techniques to determine the value of demographic, social, and psychological attributes in top management regarding success in entrepreneurial ventures. Top management members are those who are responsible for making decisions for critical affairs, whether in public or private sectors. Psychological states have been used as an important variable in research to determine whether higher managers are viable for the critical decision-making procedures meant to steer the firm toward the operational objective (Zhao and Seibert, 2006). Managers exhibit different individual attributes, some of which provide powerful motive toward progressive strategic initiatives. An institution and its strategic partners, therefore, have a role to identify, distinguish, and select the most specific managerial attributes in potential employees or human resource that resonate with their strategic requirements for entrepreneurial goals.

Green accounting practices are one of the critical decisions of strategic adoption that top management might encounter in the new business age. For most firms, long-term success means that sustainable goals have to be incorporated as important strategies to meet the dynamics of environmental challenges, and improve public perception, which is the essence of competitive advantage. Thus, increasingly, the largest impacts of top management attributes have been evaluated with the effects these may have on broader social and environmental responsibility. Many companies have been focusing on improving proactive corporate and environmental responsibility as a competitive advantage, thus orienting their leadership structure to meet such expectations (Lee et al., 2018). Corporations must, therefore, utilize green accounting as a high governance mechanism that accounts for social and environmental benefits, besides financial achievement (Lee et al., 2018).

In addition to managerial attributes, sustainable and society-oriented business practices need to be implemented under the function of interorganizational collaborations. Public-private partnership (PPP), namely the cooperation between government and social capital, is such a project operation mode in public infrastructure (Buso and Stenger, 2018). Discussion of the interwoven dynamics among top management attributes, psychological capital, and green accounting adoption is especially suitable in the context of PPP. Under a PPP model of governance, private organizations are encouraged to cooperate with the government and participate in the construction of public/private infrastructure/affairs. Due to the increasingly changing environment, civil society cooperates with the private sector and government to achieve sustainable development. PPP is considered to be a valid alternative to traditional public policies, because the use of PPPs leads to higher performances and allows governments to overcome incomplete contracts.

PPP is an important theme in the environmental protection industry. It optimizes the profitability and operation ability of listed companies from multiple aspects. Environmental PPP projects are favored by enterprises, governments, and social capital. The private sector can take advantage of adapting to the dynamic changes in environmental strategies to build a competitive edge. Such a proactive corporate role requires the organizations to harmonize internal and external resources so that the local community can meet the environmental expectations from the green practices of the business. For instance, the PPPs of energy sectors were developed with the intention of promoting energy-efficient technologies for housing, appliances, schools, commercial and public buildings, vehicles, and so on, for the development of advanced energy-efficient and low-carbon technologies and cost sharing (Jaffe et al., 2001, 2005; Sperling, 2001). The use of PPPs in climate change adaptation helps to obtain an efficient and fair allocation of risks and incentives among public and private actors (Agrawala and Fankhauser, 2008).

In sum, in the PPP context, it is important to run new initiatives such as the green accounting practices, with the positive collective mind-set (or shared cognition, holistic viewpoints, collective consensus, etc.) of decision-makers from different sectors, which is formed through their personal attributes. The aforementioned requires psychological capital as a basis of the interaction of such cross-sector interactions and collective actions. Thus, we wish to offer an in-depth articulation of the interwoven relationships between top management attributes, psychological capital, and green accounting practices adoption, which is interesting and might stimulate future thinking of research and practices.

## Theoretical Basis

For a model of personality dimensions, studies indicate various aspects of evaluating the factors and their correlating degree of impact on organizational success (Zhao and Seibert, 2006). As such, top managers are both expected to reflect differences in various personality dimensions, as well as express varying degrees of influence. For instance, managers may score high on openness to experience and conscientiousness, while expressing low agreeableness and neuroticism, as compared to entrepreneurs. They may not reflect differences in extraversion, a critical attribute required of organizational performance, but the size of the effect each personality variable presents can be small or moderate with regard to the multivariate relationship in personality dimensions.

Researchers and strategists have been studying the effects of the top management attributes on organizational performance. Essentially, leaders influence organizational dynamics with their individual attributes such as personality (e.g., Peterson et al., 2003).

## Green Accounting

One of the most fundamental goals in green accounting is the need for environmental sustainability. For sustainability, humans and nature must coexist, wherein the former keeps the value of sustainable development in mind to eradicate consequential effects of the exhaustive use of natural resources, such as hunger and poverty, while enhancing good health and well-being (Bennett and James, 2017). The PPP model optimizes the profitability and operation ability of organizations from multiple aspects, including: optimizing profitability, improving cash flow, reducing leverage on the balance sheet, improving off-balance sheet leverage, improving construction, and alleviating project payment arrears (Lopes and Caetano, 2015; Menezes and Ryan, 2015). In a PPP mode of governance, it is vital the partnership construction should be engaged together by the public sector (government) and the private sector (party) *via* projects that emphasize benefit-sharing, risk-sharing, and long-term collaboration (Bing et al., 2005; Iossa and Martimort, 2012). This mode makes up for the weakness of both the private and public sectors (Mees et al., 2012; Tompkins and Eakin, 2012).

The relationship between corporations and society is, however, so dynamic and heterogeneous that social contract alone may not characterize their interactions. Therefore, corporations adopt proactive business practices, such as the green accounting in a PPP context in line with social responsibility, to support both economic and social facets of positive environmental sustainability, and growth in society (Booth et al., 2005). As such, stakeholders will value the legitimacy of corporations in terms of how they implement environmental and social standards as instruments of their supply chain.

Numerous factors motivate the adoption of green practices and corporate environment responsibilities including stakeholder pressure, social expectations, and organizational support (Booth et al., 2005). Most importantly, social expectations shape the tendency of organizations to adopt social norms and orient their behavior to meet the required social and environmental responsibility. The resulting moral judgment creates a better understanding of obligations that best mirrors the ethical responsibility required from environmental respect.

The PPP model itself involves complex interest groups comprised of contractors, financial institutions, public sector clients, consultants and facilities management organizations (Akintoye et al., 2003; Grimsey and Lewis, 2004; Glazyrina et al., 2014; Akhmetshina and Mustafin, 2015), focusing more on social dynamics involving policy, legal and financial support, credit system construction, market operation forms of interaction, and mutual influences (Biygautane et al., 2019).

Green accounting is a branch of accounting that combines traditional accounting with natural environmental science. The main research direction of green accounting is looking toward harmony and sustainability between the economic development of enterprises and the surrounding environmental resources. Green accounting is as important as other types of accounting, and the impact of green accounting on enterprises' social resources and the environment is particularly critical. It reflects the practices that corporations undertake because of managerial decisions initiated and implemented by the top management team. The managers and their entrepreneurial advisories should analyze the environmental and social needs that resonate with organizational standards to develop an approach that takes accounting into consideration. That withstanding, top management engaged in such strategic decisions must portray the best individual and team attributes that ensure the organizations pursue environmental goals in tandem with the corporate development structure and economic projects.

### TMT Attributes and Green Accounting

Corporate environmental responsibility requires policies that shape sustainable environmental impact as well as promoting cost-effective and risk avoidance initiatives in organizational performance. The current section will identify the big five personality attributes in the top management team and attempt to propose and develop the policy models that relate each character with the appropriate approach to green practices.

The essence of organizational support for the environment is to enable society to evaluate the legitimacy of an institution’s performance with regard to set societal expectations, notwithstanding the level of economic benefits it displays, to meet the expected stakeholder standards. As such, the management has to come up with team attributes that will confer the best match for organizational performance in social and environmental responsibility. This requires leadership skills in human resource, consistent with the cultural and corporate objectives, among which must include at least one of the big five personality attributes (Zhao and Seibert, 2006). The most important team attributes that will apply in this case are conscientiousness and extraversion, which aim at developing a critical analysis of societal inadequacies to promote environmental conservative measures to adapt to dynamic changes within the environment. Besides improving employees’ perception of the company, environmental social responsibility ensures that the management team employs attributes in human resource that are committed to evaluating and allocating organizational resources to green practices that create competitive advantage (Jayanthi, 2015).

Top management’s collective attributes also need to be responsive to external environments and stakeholders. Another stakeholder-oriented policy that affects the adoption of green practices relates to the pressure mounted on the organization by parties such as local community, employees, shareholders, management, and society, who may affect a significant portion of the company’s decisions and activities. There may be some problems in the operation of the PPP model due to possible opportunistic behaviors and the differences in the target orientation between the government and the private sector. Thus, top management members need to coordinate with different external interest parties to deal with challenges (Akintoye et al., 2003). With the stakeholders’ active part in the analysis of environmental issues, they can be charged with the responsibility to identify sensitive environmental vulnerabilities and propose effective intervention measures to best meet the societal expectations, notwithstanding the economic benefits to the company. Therefore, stakeholder pressure motivates a team management spirit geared toward sustainable environmental strategy, with the fundamental goal being to contribute sustainable supply chain process in tandem with the managerial policy of green adoption practices (Lee et al., 2018). Corporations and stakeholders coordinate to create and develop sustainable goals through their effort to solicit for social, ethical, and environmental awareness on positive green accounting practices.

Also, although the government’s supervision on the private sector can enable the private sector to fulfill the contract to a certain extent, in reality, the private sector sometimes gains more additional benefits through nonperformance than the losses under the government’s supervision. In this case, the private sector will choose to risk nonperformance to obtain high benefits (Chan et al., 2011; Chiromo, 2014). To achieve sustainable development, PPP project firms may adopt the market-oriented policy as part of the corporate strategy to impact environmental performance. Customers, suppliers, government, and nongovernmental regulatory bodies have played a critical role in environmental management through social and ethical standards set to guide an organization’s code of conduct. These goals require a powerful drive to employ a personnel team that exhibits effective attributes with regard to conscientiousness, openness to experience, and extraversion as some of the big five personalities in top management team (TMT).

Green PPP practices are based on the common rules of the PPP model: compared with the traditional government responsibility system of environmental governance, the PPP model has more environmental protection responsibility and less financial pressure.

Organizations may tend to adopt green practices based on the availability of external resources linked with the top management. Under such conditions, the government can step in to promote policies that provide financial incentives intended for innovative technical resources (Rothmann and Coetzer, 2003). For companies, the government works with social capital to support their initiative to lower environmental risks. For stakeholders, the company has a role to uphold corporate governance as an important mechanism to address environmental risks, whereas the government provides voluntary environmental governance to enable institutions to adopt corporate environmental responsibility and green practices.

It is important for stakeholders and strategists to conduct environmental impact assessments to predict and manage some of the organizational activities that may negatively impact the environment. Among the assessment tools that provide a positive and synergistic effect on performance are environmental governance and supplier assessment mechanisms. The attempt to base organizational legitimacy on environmental motivation may not provide complete environmental management mechanisms, but should rather focus on competitive strategies to establish the level of effective change the organization can present in relation to other competing initiatives in the industry. Therefore, companies have the potential to affect enormous positive change in their environment, on the condition that they adopt, establish, and develop beneficial corporate and environmental initiatives.

The green initiatives require interdisciplinary coordination to affect some necessary changes in the direction and magnitude of resource allocation procedure. Therefore, human resource strategists must always endeavor to identify leadership attributes that can meet the required tacit knowledge and skills in training and develop environmentally conscious efforts (Blome et al., 2014). The leadership team and entrepreneurs in charge of innovative practices should exhibit competent skills in their openness to learning in order to advance green practice adoption, for the reason that the propensity to adopt innovative technology directly relates to becoming receptive to new environmental ideas, which requires a team of managers with an openness to learning.

Green accounting practices would, therefore, rely on useful analysis of the behaviors of top managers regarding their attitudes and behaviors. Most of the factors that influence their behaviors include workplace challenges, the pressure to achieve the required goals, and other factors attributed to personal difficulties (Shang Guan et al., 2017). Also, the environments for adopting the green accounting program could be unfavorable for the drafted strategies, thus discouraging organizational leaders. On the other hand, positive personality contributes to fast and effective growth of corporate progress. The process creates a reliable system through which the stakeholders can have a platform for adopting the right green accounting practices even with limited resources. A positively minded manager would achieve the green accounting goals by empowering other managers.

### TMT Attributes, Psychological Capital, and Green Accounting

The psychological capital is important in building a relationship between leaders and their followers (Kong et al., 2018), especially when taking on collective initiatives. It focuses on strengthening the virtues and merits of an organization (Luthans et al., 2004, 2007; Luthans and Youssef, 2004). The attitudes and behaviors of the managers tend to influence performance and value-based practices. Thus, psychological capital acts as a component that facilitates improved organizational activities for the top management to address issues linked to other forms of capital and other factors, such as behaviors of the employees (Avey et al., 2008, 2009, 2011).

In the context of PPP, institutional entrepreneurs and top management members are also “agents” of society, creating consistent technical and cognitive norms, models, scripts, and behavioral patterns (Déjean et al., 2004). In PPP projects, norms are shaped coordinating work among various organizational entities (Biesenthal et al., 2018), exploring the organizational dynamics of the project (Miterev et al., 2017). The process of institutional entrepreneurship is even far beyond the power of institutional entrepreneurs and can actively address the institutional and technical constraints that hinder PPPs (Biygautane et al., 2019). Individuals’ thoughts and actions are influenced by the institutional environment they are in, and individuals interact with each other to change the organization they lead (Battilana and D’aunno, 2009).

The primary role played by the organizational leadership is to identify the right talents in adopting the proper practices in green accounting program. However, the persecution of people matters significantly for the case that needs collective efforts for the implementation of the program (Chen et al., 2017). Also, the listed standards for developing the necessary system would ensure a useful repository guideline that would address significant leadership differences that could hinder progressive development. Green accounting in PPP contexts is one of the programs that aims to address sustainability issues across different organizations. Therefore, most of the strategies have focused on sustainable goals that would reduce the negative environmental impacts of different infrastructural activities. As a result, the administrative functions within the organization should consider the constraints limiting people’s strengths and talents from prospering in such a workplace setting where green accounting may function well.

Since the success all depends on key persons and their interactions as mentioned above, a positive mind-set and the encouragement of development projects would have a vital impact. In such a premise, psychological capital facilitates effective programs toward developing such sustainable leadership as discussed here. Such leadership would benefit the management of people with the aim of improving the right platform for adequate delivery of sustainable services (Chen et al., 2017).

Stable psychological attributes contribute to long-term characteristics of the organization. The fluid attributes are common in most organizations. However, they are considered less as future predictors since they regularly change. A similar case applies to personal values and the consequent actions they would have, as well as forming a positive working culture. Behaviors and attributes vary based on significant psychological factors associated with such multiple interest parties. Therefore, most of the attitudes presented by the employees or leaders have less impact on the future progress of the organizational programs, unless the collective psychological capital functions with impacts.

## Conclusion and Implications

Leaders such as the top management in a PPP context need to reflect charismatic, visionary, inspirational attributes to affect society positively. In contrast, ineffective leaders have particular negative attributes related to irritable, noncooperative, and egocentric indulgence, which amount to dictatorial leadership. Companies and their respective government policies must, therefore, relate institutional corporate strategies with individual behaviors to promote positive leadership.

To this effect, scholars developed a cross-sector interfirm knowledge framework to enhance organizational learning and new practice adoption, which can be resource-based, competence-based, or knowledge-based. For instance, they need to have a model of addressing the employees’ challenges and various contexts of conflicting behaviors. Attention should focus on developing a fair system that treats its employees fairly. The selected attributes are shown to reflect on the performance of employees across different occupational groups. Therefore, this impacts differently on the ability of the leaders to develop the right governance programs aimed at addressing sustainability issues. Although not sufficient to guarantee success, effective leadership must develop elaborate structures to support the interorganizational challenges, as well as to ensure consistency of best practices. Those factors that underscore good leadership in connecting collaborative organizations include the selection and reelection of merit leadership, performance management, establishment, and evaluation of ethical standards, talent development, and retention, development of strategic priorities, wide cross-sectional stakeholder engagements, and independent and effective evaluations, among other supportive leadership concerns.

The top management attributes reflected the social and psychological expectations that people have regarding working with other people across organizations. Discussion for social psychological issues, such as this paper engages in, contributes to the identification of the right behaviors to develop collaborative success. Green accounting focuses on developing a reliable system that balances multiple-party interests. Supported by this concept, all parties involved in PPP are based on the spirit of contract and good faith, supplemented by excellent financial and market environments, and social capital actively participates in infrastructure and public services such as green accounting. Such a cooperation mechanism is bound to bring out the best economic and social benefits.

## Author Contributions

C-CC and YJ were the major contributors and writers of the first manuscript. H-YL edited the manuscript and was in charge of the revision & resubmission (R&R) writing process. Y-TL conceptualized and validated the article and co-wrote in the R&R stage.

### Conflict of Interest Statement

The authors declare that the research was conducted in the absence of any commercial or financial relationships that could be construed as a potential conflict of interest.
